# Bistatic Radar with Quantum-Generated Noise Phase Manipulation and Non-Directional Antennas

**DOI:** 10.3390/s26051717

**Published:** 2026-03-09

**Authors:** Nikolay Gueorguiev, Atanas Nachev, Ognyan Todorov, Tereza Trencheva, Gergana Chalakova

**Affiliations:** 1 Institute of Robotics, Bulgarian Academy of Sciences, Acad. Georgi Bonchev Str., Bl. 2, 1113 Sofia, Bulgaria; niki0611@abv.bg; 2 Faculty of Information Sciences, University of Library Studies and Information Technologies, 119, Tsarigradsko Shosse Blvd., 1784 Sofia, Bulgaria; nachew@abv.bg; 3 Technopol Ltd., 6000 Stara Zagora, Bulgaria; odt@technopol.bg; 4 Faculty of Library Studies and Cultural Heritage, University of Library Studies and Information Technologies, 119, Tsarigradsko Shosse Blvd., 1784 Sofia, Bulgaria; t.trencheva@unibit.bg; 5 Institute of Mechanics, Bulgarian Academy of Sciences, Acad. Georgi Bonchev Str., Bl. 4, 1113 Sofia, Bulgaria

**Keywords:** bistatic radar, quantum generation of digital random numbers, random phase manipulation, controlled electromagnetic center, trilateration

## Abstract

**Highlights:**

**What are the main findings?**

- A quantum-generated random number sequence approach for phase manipulation control of bistatic noise radars is proposed.
- A method for correlation-based processing of the received signals is proposed in which the emissions from both antennas are phase-controlled to arrive either in phase or out of phase at a predetermined point in space.

**What are the implications of the main findings?**

- A bistatic radar with random phase manipulation is developed based on an infinite, non-repeating digital code.
- Radar signal processing capability and emission concealment are improved through controlled resultant electromagnetic field generation.

**Abstract:**

The development of bistatic noise radars is a promising contemporary direction in the field of radar technology. Two novel approaches are proposed in this study as further development of existing methods for their design. The first approach involves using a quantum-generated random number sequence for phase manipulation control, which is practically infinite in duration. This ensures additional electronic protection of the radar, since the phase manipulation control code will not repeat regardless of the duration of its operation. The second approach is related to the introduction of synchronized emissions from both antennas in a manner ensuring equality or controlled difference of their signals upon arrival at a predetermined point in space. This enables the formation of a controlled electromagnetic field. As a result, received-signal processing capabilities are improved, while additional electronic “stealth” is achieved by creating a fictitious electromagnetic center of the radar’s resultant radiation (i.e., an effective RF phase center of the resultant emission) and complicating the determination of antenna locations. A block diagram and general algorithm for information processing of a bistatic radar with quantum-generated noise phase manipulation and non-directional antennas are proposed in this study.

## 1. Introduction

Due to their ability to detect small-sized and low-flying aerial objects, multistatic and particularly bistatic radars are being increasing used. Bistatic radars are radar systems in which the transmitter and receiver are located at different positions (unlike monostatic radars, where they are co-located). They have specific advantages and disadvantages [1,2,3,4,5,6]. The main advantages of bistatic radars include lower vulnerability to electronic countermeasures (ECMs) and improved detection of low-observable (“stealth”) targets. Additional benefits are enhanced deployment flexibility according to terrain and specific tasks, suitability for network-centric configurations, multi-position configurations for radar surveillance, and increased reliability and survivability. The main disadvantages of bistatic radars are their more complex synchronization system and hardware and signal processing, their higher cost at the system level, and the need for communication channels between elements. Bistatic radars generally consist of receiving and transmitting components located at different points in space, where the radiation patterns of the transmitting and receiving components can be directional or non-directional. The use of directional patterns provides the possibility of determining the three coordinates of objects detected by the radar, but the radiated electromagnetic energy is not fully utilized. Moreover, placing only a transmitting (Transmitter—Tx) or a receiving (Receiver—Rx) component at a given point in space is more expensive than co-locating receiving and transmitting components in a single integrated technical structure positioned at one location. This is why, in many cases, it is suggested to deploy multiple transmitters and receivers as a network of bistatic radar links, where each transmitter–receiver combination constitutes its own bistatic channel [1,2,3,4]. Obviously, bistatic radars have a specific area of application, primarily in combination with classical monostatic radars. Despite monostatic radars being widely used, in recent years, research related to various approaches for creating bistatic radars, including those using random manipulation of transmitted signals, has been growing [7,8,9,10,11,12,13,14,15,16,17,18,19,20,21]. The topic “bistatic radar” has over 14,000 scientific publications, and in the past five years, the number of publications on “bistatic radar” indexed in WoS/SciVerse and Scopus has ranged from 200 to over 330 annually, of which up to 4 publications per year directly discuss randomized/phase-manipulated modulation in bistatic radar configurations. This trend shows increased interest in the scientific community on this topic and moderate but steady growth of research on bistatic radars. In parallel, the past decade has seen significant development of quantum systems for various purposes [22,23,24,25,26,27]. Quantum communication technologies create new opportunities for their application in the field of radar technology [28,29,30,31,32,33]. One of the characteristic features of radars, including bistatic radars using random phase manipulation, is their continuous operation over extended periods of time—across days, weeks, and even months. In this case, problems related to their stealth arise for two reasons. The first is because during prolonged operation, the random key that controls the phase manipulation begins to repeat, which allows for its disclosure and the organization of effective ECM. Modern cryptographically secure pseudo-random number generators (CSPRNGs), such as AES-CTR-DRBG, ChaCha20-based CSPRNGs, Fortuna, NIST DRBG constructions, etc., are capable of producing practically non-repeating codes, especially when the code is periodically reseeded to ensure phase stability while maintaining a limited coherent processing interval (CPI). When reseeding is applied, the newly generated code does not maintain phase continuity with the previous one; each code segment remains internally coherent. The use of a shortened code combined with periodic reseeding does not degrade correlation performance, provided that the reseeding is synchronized and performed only between coherent processing intervals. In this context, the use of QKD represents an additional mechanism for secure reseeding, particularly in cases where a quantum communication system is already deployed between the two branches of the bistatic radar. Furthermore, QKD facilitates synchronized and controlled reseeding, enables the CPI to be kept below the atmospheric coherence time (especially when the CPI is limited but the mission duration is long), reduces internal decorrelation, and supports adaptive digital phase compensation. The second reason is that prolonged radar operation enables the location of the transmitting antennas to be determined. These risks are particularly high in cases of bistatic radars with continuous and non-directional emission, where the radiated electromagnetic energy and the positioning of the bistatic radar components can be utilized to the maximum extent. Therefore, the main objective of the present research is to find a possibility for reducing the abovementioned problems by using quantum technologies.

## 2. Materials and Methods

### 2.1. General Approach

In the present study, the possibilities for creating a bistatic radar with non-directional emission, a practically non-repeating random code for signal phase manipulation, and protection against electronic determination of the locations of its transmitting antennas are examined. This study is relatively complex and may be considered fundamental for the development of other configurations of networked multistatic radar systems.

A bistatic radar generally consists of two transmitting and two receiving units, and each receiver processes echoes from both transmitters [34,35,36,37,38]. As a result, each Tx − Rx pair forms an independent radar channel, i.e., the described bistatic radar (of the 2Tx − 2Rx type) will have four bistatic channels (${\mathrm{Tx}}_{1}{\mathrm{Rx}}_{1}$, ${\mathrm{Tx}}_{2}{\mathrm{Rx}}_{1}$, ${\mathrm{Tx}}_{1}{\mathrm{Rx}}_{2}$, and ${\mathrm{Tx}}_{2}{\mathrm{Rx}}_{2}$). Each channel has its own bistatic geometry, delay, Doppler shift, and phase.

One method for creating a practically non-repeating random code for signal phase manipulation is the use of quantum generators for its formation. To enable each receiver to process echo signals from both transmitters, the codes controlling the phase manipulations in the two transmitters must be identical. This can be ensured by using keys generated within a quantum communication system [22,23,24,25,26,27].

To hinder the electronic determination of the transmitting antenna locations, it is effective to apply a method for creating a fictitious or controllable electromagnetic center of the resultant emission in addition to using a noise signal [39,40].

A suitable means for combining the two methods—ensuring a practically non-repeating random code and creating a controllable electromagnetic center—is using the bistatic radar Quantum System for Creating Random Phase-Manipulated Emissions with Controlled Electromagnetic Center [41] as a transmitting center.

In quantum key distribution (QKD), two communicating parties generate a random key at a distance through a specific procedure called a QKD protocol, whose randomness and security are guaranteed by the properties of the quantum system and by the fact that quantum states cannot be copied [22,23,24,25,26,27].

To ensure synchronization between the key generation and update rates in the Quantum System for Creating Random Phase-Manipulated Emissions with Controlled Electromagnetic Center [41] and the phase manipulation rate required for the bistatic radar, it is proposed to use pseudo-random number generators (PRNGs) with a controllable number of generated values equal to *N*, whose seed inputs are connected to the outputs of the Key Management Systems (KMS) of the corresponding channels of the QKD system.

Accordingly, the quantum system exploits the generated keys to form a practically infinite random sequence for controlling a digital phase manipulator and to allow for control of the main operating frequency (command *f*), variation in the length of the generated random sequences through which phase manipulation is implemented (command *N*), synchronous resetting of initial phases, and controlled dephasing of the generated signals (commands $f_{0}$, $\Delta f_{01}$, $\Delta f_{02}$) and time shifts $\Delta T_{1}$ and $\Delta T_{2}$. In this manner, similarly to MIMO radar systems, synchronization of the two channels is ensured, enabling the simultaneous arrival of the radiated RF signals at a predetermined point in space with a controlled in-phase relationship.

The system also provides the possibility for correcting the amplification of the radiated signals from the two channels ($\Delta P_{1}$ and $\Delta P_{2}$), compensating for the different distances between the antennas of the two channels and the specified point where the signals must be equal or have a controlled difference.

Synchronization between the two channels  [41] may be achieved using a satellite positioning system (GNSS); however, to ensure autonomy from navigation systems, it is recommended to use a specialized White Rabbit timing channel (IEEE 1588v2 + SyncE) with accuracy below 1ns or an optical time-transfer channel providing a synchronized clock [42,43,44,45,46,47].

Through the control of the generated signals and synchronization of the quantum system, it is possible to ensure a stable and synchronized reference signal for operating the receiving centers of the bistatic radar. On this basis, a block diagram and a general algorithm for information processing of a Bistatic Radar with Quantum-Generated Noise Phase Manipulation and Non-Directional Antennas can be synthesized.

### 2.2. System Implementation

**Figure 1:**
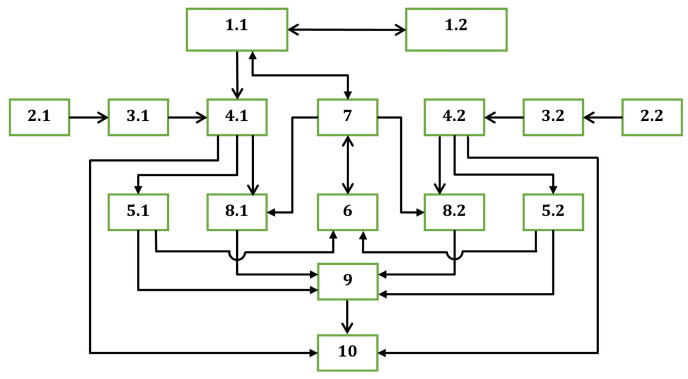
Block diagram of Bistatic Radar with Quantum-Generated Noise Phase Manipulation and Non-Directional Antennas.

Based on the above, it is possible to create a Bistatic Radar with Quantum-Generated Noise Phase Manipulation and Non-Directional Antennas (Figure 1), which consists of two components connected by a quantum communication system, located at different points in space, and a joint Command Center and additional modules for processing information from the two components.

The notations used in Figure 1 are as follows:

- Transmitting channels (1.1,1.2);
- Receiving antennas (2.1,2.2);
- Wideband controllable amplifier modules (3.1,3.2);
- Correlation receivers (4.1,4.2);
- Amplitude threshold exceedance selection modules (5.1,5.2);
- Module for determining potential target coordinates (6);
- Command Center (7);
- Amplitude non-exceedance selection modules (8.1,8.2);
- Module for determining probable target coordinates (9);
- Final analysis module (10).

Each of the components has identical transmitting and receiving channels, while the joint Command Center and additional information processing modules can be located in one of the two components of the bistatic radar or in a separate facility.

The transmitting channels (1.1,1.2) are implemented based on the Quantum System for Creating Phase-Manipulated Emissions with Controlled Electromagnetic Center, and according to its design, one of the channels (1.1) is the master.

As previously noted [41], the Quantum System for Creating Phase-Manipulated Emissions with Controlled Electromagnetic Center allows for control of the operating frequencies, frequency and phase offsets in the two channels, and phase manipulation periods, and it can ensure equality or controlled difference of the signals from the two system channels upon their arrival at a predetermined point in space with coordinates $\left(X_{i},\;Y_{i},\;Z_{i}\right)$.

The two receiving channels are identical, and each is located in one housing with one of the transmitting channels and includes receiving antennas (2.1,2.2) connected to the inputs of wideband controllable amplifier modules (3.1,3.2), whose operating frequency control inputs are connected to the operating frequency control outputs of the corresponding transmitting channels (1.1,1.2). The outputs of the wideband controllable amplifier modules (3.1,3.2) are connected to the signal inputs of correlation receivers (4.1,4.2), whose reference inputs are connected to the transmitting channels (1.1,1.2). The correlation receivers’ outputs are connected to additional modules for processing received information and for emission control to eliminate false targets.

The additional modules for processing received information and for emission control to eliminate false targets consist of amplitude threshold exceedance selection modules (5.1,5.2) connected to the outputs of the correlation receivers (4.1,4.2). These modules register correlation functions exceeding a predetermined threshold and determine the corresponding delay times. The delay corresponds to the probable distance to the sources of echo signals, which in general is the radius of the hemisphere of possible target location [48,49,50].

Due to the signals of the two transmitting channels (1.1,1.2) being of similar nature, in general, for one and the same real target, each of the correlation receivers (4.1,4.2) will generate data for two types of hemispheres of possible target locations. The first type of hemisphere, referred to as true, is determined by the delay time of the signal reflected from the target of the transmitting channel co-located with the correlation receiver (i.e., from correlation receiver (4.1) for a reflected signal from (1.1), or from correlation receiver (4.2) for a reflected signal from (1.2)). The second type of hemisphere, referred to as false, is determined by the delay time of the signal reflected from the target of the transmitting channel located in a different housing from the correlation receiver (i.e., from (4.1) for a reflected signal from (1.2) or from (4.2) for a reflected signal from (1.1)).

The amplitude threshold exceedance selection modules (5.1,5.2) of the two receiving channels are connected to the inputs of the module for determining potential target coordinates (6). In this module, the task of finding the coordinates where the two types of hemispheres intersect is completed. These coordinates include those resulting from the intersection of true hemispheres, as well as multiple coordinates resulting from the intersection of false and true hemispheres or false and false hemispheres. Thus, even with only one real target, intersections of a total of four hemispheres (two true and two false) may result in up to eight possible target coordinates. Two of these coordinates result from the intersection of true hemispheres, with one corresponding to the actual target location and the other being its mirror image.

To eliminate the coordinates of false targets, it is initially proposed to use the capability of the Quantum System for Creating Phase-Manipulated Emissions with Controlled Electromagnetic Center to control the operating frequencies, frequency and phase offsets in the two channels, and phase manipulation periods to ensure equality or controlled difference of the signals from the two system channels upon their arrival at a predetermined point in space with coordinates $\left(X_{i},\;Y_{i},\;Z_{i}\right)$. For this purpose, the coordinates from the output of the module for determining potential target coordinates (6) are sent to the Command Center (7), which is connected to the control input of the Quantum System for Creating Phase-Manipulated Emissions with Controlled Electromagnetic Center to ensure equality with periodic phase switching (in-phase and out-of-phase relationships) of the signals from the two channels upon their arrival at a point in space corresponding to the coordinates $\left(X_{i},\;Y_{i},\;Z_{i}\right)$ of the analyzed potential target.

If these coordinates result from the intersection of true hemispheres, then in the out-of-phase case, the echo signals arriving at the real target will mutually compensate and the amplitude at the outputs of the correlation receivers (4.1,4.2) will be minimal, whereas in the in-phase case, this amplitude will be maximal. To utilize this phenomenon, the outputs of the correlation receivers (4.1,4.2) are also connected to the inputs of amplitude non-exceedance selection modules (8.1,8.2), whose gating inputs are connected to the timing gate control outputs of the Command Center (7), and the selection modules are only activated during the time interval in which signal reception from a target with coordinates $\left(X_{i},\;Y_{i},\;Z_{i}\right)$ is expected.

The outputs of the amplitude non-exceedance selection modules (8.1,8.2) and the amplitude threshold exceedance selection modules (5.1,5.2) are connected to the inputs of the module to determine probable target coordinates (9). In this module, recognition of zones where true hemispheres intersect is performed if, in the out-of-phase case, the signals at the outputs of the correlation receivers (4.1,4.2) are close to zero and do not exceed the thresholds of the amplitude non-exceedance selection modules and if, in the in-phase case, the signals at the outputs of the correlation receivers exceed the thresholds of the amplitude threshold exceedance selection modules. The remaining coordinates of intersecting hemispheres are considered invalid, and the false hemispheres causing them are eliminated.

The output of the module for determining probable target coordinates (9) and the outputs of the correlation receivers (4.1,4.2) are connected to the inputs of the final analysis module (10), where selection of false targets, including mirror-image target coordinates, is performed through analysis of Doppler components of the signals, reliability analysis based on hemisphere radii, trajectory-based information processing, analysis of expected directions and trajectories of possible targets, and other methods, including the use of artificial intelligence or an operator.

The output of the final analysis module (10) is connected to the user of bistatic radar information.

It is also possible to tune and calibrate the bistatic radar, whereby through the Command Center (7), the coordinates of a reference reflector $\left(X_{0},\;Y_{0},\;Z_{0}\right)$ are set and commands are sent programmatically or manually to adjust the control of operating frequencies, frequency and phase offsets in the two channels, and phase manipulation periods, so that the signals received from the reflector at the outputs of the correlation receivers (4.1,4.2) are maximal in the in-phase case and minimal in the out-of-phase case. The reference reflector can be stationary or a mobile object transmitting its coordinates $\left(X_{0},\;Y_{0},\;Z_{0}\right)$ in real time with high accuracy.

## 3. Results

**Figure 2:**
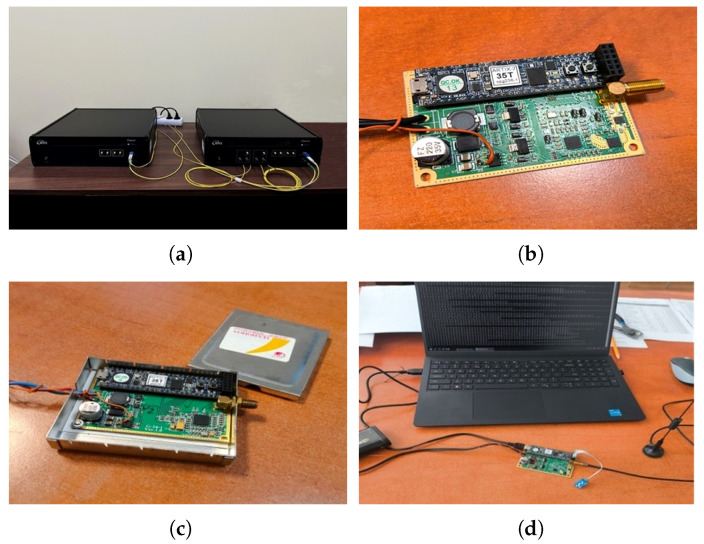
External view of the main modules of the laboratory model of a bistatic radar with quantum-generated noise phase manipulation and omnidirectional antennas: (**a**) quantum communication module based on the ID Quantique Clavis3 RND platform; (**b**) laboratory model of the quantum system for creating phase-manipulated emissions with a controlled electromagnetic center; (**c**) receiving channel Rx; (**d**) programming, control, and system management interfaces.

Based on the block diagram shown in Figure 1, an algorithm was developed and a specialized software product was implemented to perform the specified operations. A laboratory model of the main subsystems of a Bistatic Radar with Quantum-Generated Noise Phase Manipulation and Non-Directional Antennas was realized (Figure 2). The model consists of a laboratory model of a Quantum System for Creating Phase-Manipulated Emissions with a Controlled Electromagnetic Center, incorporating two identical transmitting channels (1.1 and 1.2) denoted as ${\mathrm{Tx}}_{1}$ and ${\mathrm{Tx}}_{2}$, positioned at a fixed distance from each other, and a laboratory model of a single receiving channel denoted as Rx.

In the laboratory model of the Quantum System for Creating Phase-Manipulated Emissions with a Controlled Electromagnetic Center, a quantum communication module based on an RND platform manufactured by ID Quantique was used (Figure 2a). The platform used was Clavis3, operating under the Coherent One Way (COW) protocol.

The system requires four optical communication channels: one quantum channel, two unidirectional service channels, and one classical communication channel. Optical interconnections were implemented using single-mode fiber patch cables with LC/UPC–LC/UPC duplex connectors. Key management was performed using a software-based Key Management System (KMS) developed by ID Quantique, with communication between the KMS and the remaining equipment carried out in accordance with the etsi014 protocol.

The laboratory models of the remaining modules of the Quantum System for Creating Phase-Manipulated Emissions with a Controlled Electromagnetic Center (Figure 2b) and of the receiving channel Rx (Figure 2c) were implemented as FPGA-based processing modules using the CMOD-A7-35T platform, incorporating the developed software and interfaces for programming, control, and system management (Figure 2d). Non-directional monopole-type antenna systems, RF power supply modules, and auxiliary equipment were employed.

The transmitted signal power was set to 10 mW, with an operating frequency of 1 GHz, corresponding to a wavelength of approximately 0.3 m.

The employed chip rate is 10 Mcps (megachips per second), the code length is 8192 chips, and the coherent processing interval (CPI) is 0.8 ms. Under these conditions, an approximate correlation processing gain of ≈39 dB is achieved. The radar range resolution is determined by the width of the correlation peak. For the laboratory prototype, the theoretical range resolution corresponding to the effective signal bandwidth (10 MHz) is approximately 15 m. The practical localization accuracy depends on the signal-to-noise ratio (SNR) after correlation processing and on the effective narrowing of the correlation peak under high-SNR conditions. In the laboratory experiments, the post-correlation SNR exceeded −20 dB at the receiver input, and with a processing gain above 30 dB, a reflector located at 10 m was detected with an accuracy better than 1 m.

No output power compensation was applied to account for differences in propagation paths. The quantum communication link between ${\mathrm{Tx}}_{1}$ and ${\mathrm{Tx}}_{2}$, as well as the connection between the receiving channel Rx and the first transmitting channel ${\mathrm{Tx}}_{1}$, were implemented using optical fiber.

A key aspect in the development of the laboratory model was the software implementation of the correlation receiver. In the considered radar configuration, the transmitted signal is phase-manipulated using a noise-like code sequence that is practically non-repeating in time due to continuous updating of the control codes. Although the code has a random character, it is known and synchronized between the transmitter and the receiver, which enables coherent correlation processing.

In the receiver, a local reference copy of the phase-manipulated signal is generated using an identical code sequence and synchronization in time and phase. The received echo signal, containing delayed and attenuated reflections from the target, is multiplied by the local reference copy and integrated over a predefined time interval. When the locally generated code matches the code embedded in the echo signal, the correlation function reaches a maximum, resulting in a sharp increase in the receiver output signal.

The practically infinite and non-repeating code sequence does not impede correlation reception since signal processing is performed over a finite time window determined by the integration length and the expected delay interval. Within this window, the code is fully deterministic for the legitimate receiver but remains statistically random for an external observer. As a result, noise, interference, and non-matching reflections accumulate in an uncorrelated manner and are suppressed, whereas the useful echo signal is enhanced proportionally to the integration time.

To implement the correlation receiver within the receiving channel Rx, a directional coupler was employed to extract a reference signal directly from the already generated transmitted signal in the first transmitting channel ${\mathrm{Tx}}_{1}$ prior to power amplification (direct reference approach). This ensures near-perfect frequency and phase identity between the reference and transmitted signals, requiring only compensation of the internal signal delay between the directional coupler, the transmitting antenna, and the correlation receiver. In the laboratory setup, a Pulsar Microwave CCS30-01-437/3A-type directional coupler was used.

The reference signal of the correlation receiver was subjected to I/Q down-conversion, digitized, and stored in memory (FIFO/RAM), after which it was used with an adjustable delay for comparison with the digitized input signal received by Rx.

The laboratory model was developed using open-source software tools for software-defined radio (SDR) and signal processing, specifically GNU Radio. FPGA IP configuration (matched filter and correlator cores) was performed using the AMD Vivado software suite, an integrated development environment for synthesis, analysis, and implementation of VHDL-based designs targeting Xilinx FPGA devices.

The development process proceeded through several stages, including the creation of an SDR software prototype (USRP/Pluto) in GNU Radio with code validation and delay compensation, followed by FPGA configuration, I/Q ADC integration, FIFO and RAM implementation, and final deployment of an FFT-based correlator IP core together with a dedicated calibration module. RF and I/Q conversion were performed outside the FPGA, while two complex I/Q streams were fed into the FPGA: ${\mathrm{REF}}_{I/Q}$ from ${\mathrm{Tx}}_{1}$ via the directional coupler, and ${\mathrm{Rx}}_{I/Q}$ from the receiving channel.

For experimental purposes, compensation of the internal signal delay between ${\mathrm{Tx}}_{1}$ and Rx was performed deterministically at the beginning of the experiments by injecting a calibration pulse as a direct signal from ${\mathrm{Tx}}_{1}$ to Rx. The resulting calibration correlation maximum corresponds to the internal system delay, which was subsequently subtracted from the correlation maxima obtained during actual measurements.

**Figure 3:**
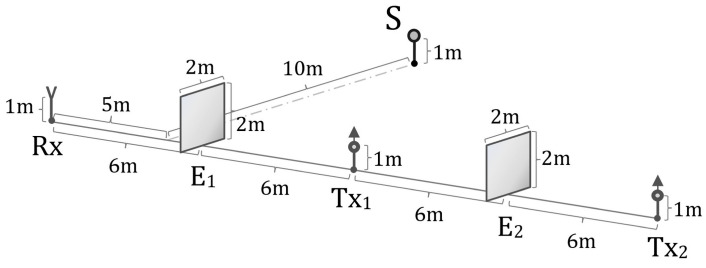
Schematic of the experimental setup showing the arrangement of the transmitting channels (${\mathrm{Tx}}_{1}$, ${\mathrm{Tx}}_{2}$), receiving channel (Rx), shielding screens ($E_{1}$, $E_{2}$), and spherical reflector (S).

To suppress direct signal leakage from the transmitting channels to the receiver, shielding screens denoted as $E_{1}$ and $E_{2}$ were installed between ${\mathrm{Tx}}_{1}$, ${\mathrm{Tx}}_{2}$, and Rx. The experimental arrangement is shown in Figure 3. A metallic spherical reflector with a radius of 0.30m, denoted as S, was used as the target. The components ${\mathrm{Tx}}_{1}$, ${\mathrm{Tx}}_{2}$, Rx, $E_{1}$, and $E_{2}$ were positioned along a straight line with mutual distances of 6m between adjacent elements. The reflector was located at a distance of 10m from this line. The antenna heights and the height of the spherical reflector were identical and equal to 1m. The shielding screens were steel plates with a thickness of 0.2cm and dimensions of 2 × 2m, grounded using three metal grounding plates each 5mm thick. Due to their mass of approximately 63kg, the screens were mounted horizontally using a square-profile frame and fixed with twelve bolts per plate.

Distance measurements were conducted using a Huepar LM100A laser rangefinder (100 m range) with a measurement accuracy of 1.6mm. RF power measurements were carried out using a wideband RF power sensor, NRP-Z81 (Rohde & Schwarz, Tokyo, Japan), connected to an R&S FSH-20 spectrum analyzer, enabling measurement of short-duration signal sequences with temporal structures down to 100ns. The correlation function was measured at the output of the correlation receiver.

The averaged measured signal power at the location of the spherical reflector from a single transmitting channel, obtained over ten consecutive measurements, was approximately $8.6\times 10^{-8}\;W$ (86nW). The averaged total power from both transmitting channels, measured over twenty consecutive runs without ensuring phase alignment at this point, was $1.7\times 10^{-7}\;W$ (170nW). The maximum total power from both transmitting channels, measured over twenty consecutive runs with ensured in-phase operation, reached $3.5\times 10^{-7}\;W$ (350nW).

The averaged values of the correlation function over twenty consecutive runs were close to unity (0.978) when the emissions were phase-aligned at the reflector location and close to zero (0.035) in the out-of-phase case. These results confirm the feasibility of a bistatic radar with quantum-generated noise phase manipulation and a practically infinite random code, employing non-directional antennas to generate a controllable electromagnetic field that can form a controllable electromagnetic center and spatial zones of constructive (in-phase) and destructive (out-of-phase) superposition of the emitted signals.

## 4. Discussion

For a general assessment of the feasibility and operational characteristics of the proposed bistatic radar block diagram, it is necessary to analyze the phenomena associated with the propagation of two coherent electromagnetic waves generated by spatially separated sources, as well as the reflected signals received at two spatially separated receiving centers.

As is well known, for two coherent non-directional (isotropic) sources of electromagnetic waves located at a certain distance from each other, the interference pattern is determined by the difference in the propagation paths of the waves from the two sources to each point in space. Due to the wave nature of the emitted signals, under coherent emission, the interference pattern represents a sequence of interference waves with maxima (when the two signals are in phase) and minima (when the two signals are out of phase), taking the form of two-sheet hyperboloids of revolution with foci located at the positions of the two emitting sources [48,49,50]. This interference pattern constitutes a system of surfaces that are symmetric with respect to the line connecting the two sources and are nested within each other; when intersected by a plane, hyperbolas are obtained.

When dephasing the phase-manipulated emissions and introducing delays in the moments of phase switching in a manner ensuring in-phase operation at a point $T_{i}$ with coordinates $\left(X_{i},Y_{i},Z_{i}\right)$, the shape of the interference pattern changes such that one of its maxima passes through this point. Consequently, by changing the location of the point (i.e., its coordinates $\left(X_{i},Y_{i},Z_{i}\right)$), controlled modification of the interference pattern of the emitted signals, and accordingly, of the equivalent electromagnetic center of emission, is achieved.

The difference between the distances from the two transmitters to the point at which in-phase operation (or controlled dephasing) of their emissions is intended to be achieved determines the required values of the main controllable parameters of the proposed bistatic radar—namely the phase offsets $\Delta f_{1}$, $\Delta f_{2}$, the time delays of the applied clock pulses causing phase manipulation $\left(\Delta T_{1},\Delta T_{2}\right)$, and the output power corrections of each channel $\left(\Delta P_{1},\Delta P_{2}\right)$.

**Figure 4:**
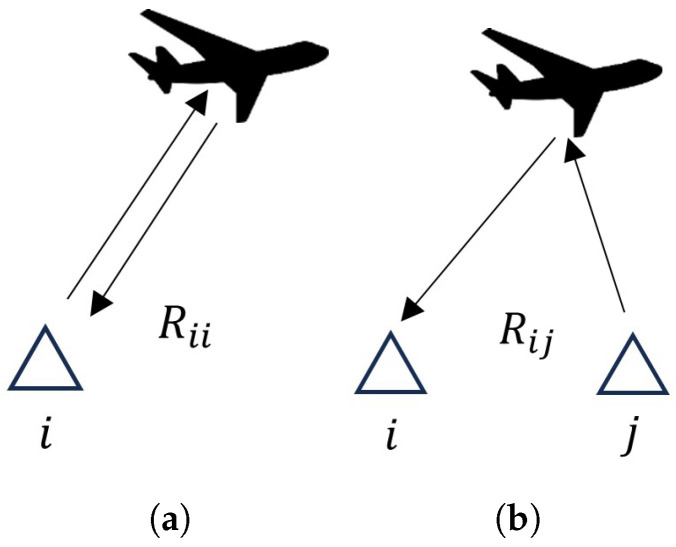
Real and fictitious distances to the aircraft when creating a fictitious electromagnetic center: (**a**) real distance; (**b**) fictitious distance.

Considering the fact that the two transmitting devices emit identical signals, when the method for creating a fictitious electromagnetic center is used, each *i*-th receiving device receives several echo signals: one reflected from the signal emitted by its own transmitter, and the others reflected from radiated signals by one of the remaining *j*-th transmitters. On this basis, the *i*-th receiver determines several distances $R_{ij}$ to the aircraft (Figure 4). One of these distances, $R_{ii}$, is real (Figure 4a), while the remaining distances $R_{ij}$(j≠i) are fictitious (Figure 4b).

In principle, the distances to the aircraft define its possible location on the surface of a hemisphere situated above the Earth’s surface with radius $R_{ij}$. Since, on the one hand, $R_{ij}=R_{ji}$, and on the other hand, the differences between $R_{ji}$ and $R_{jj}$ and between $R_{ij}$ and $R_{ii}$ are equal, all fictitious distances to the aircraft can be eliminated in the block for combining information from multiple receiver units/RX modules. After this elimination, the aircraft location can be determined using the trilateration method [48,49,50], while it is advisable to take into account the uncertainty of information in angular coordinates.

**Figure 5:**
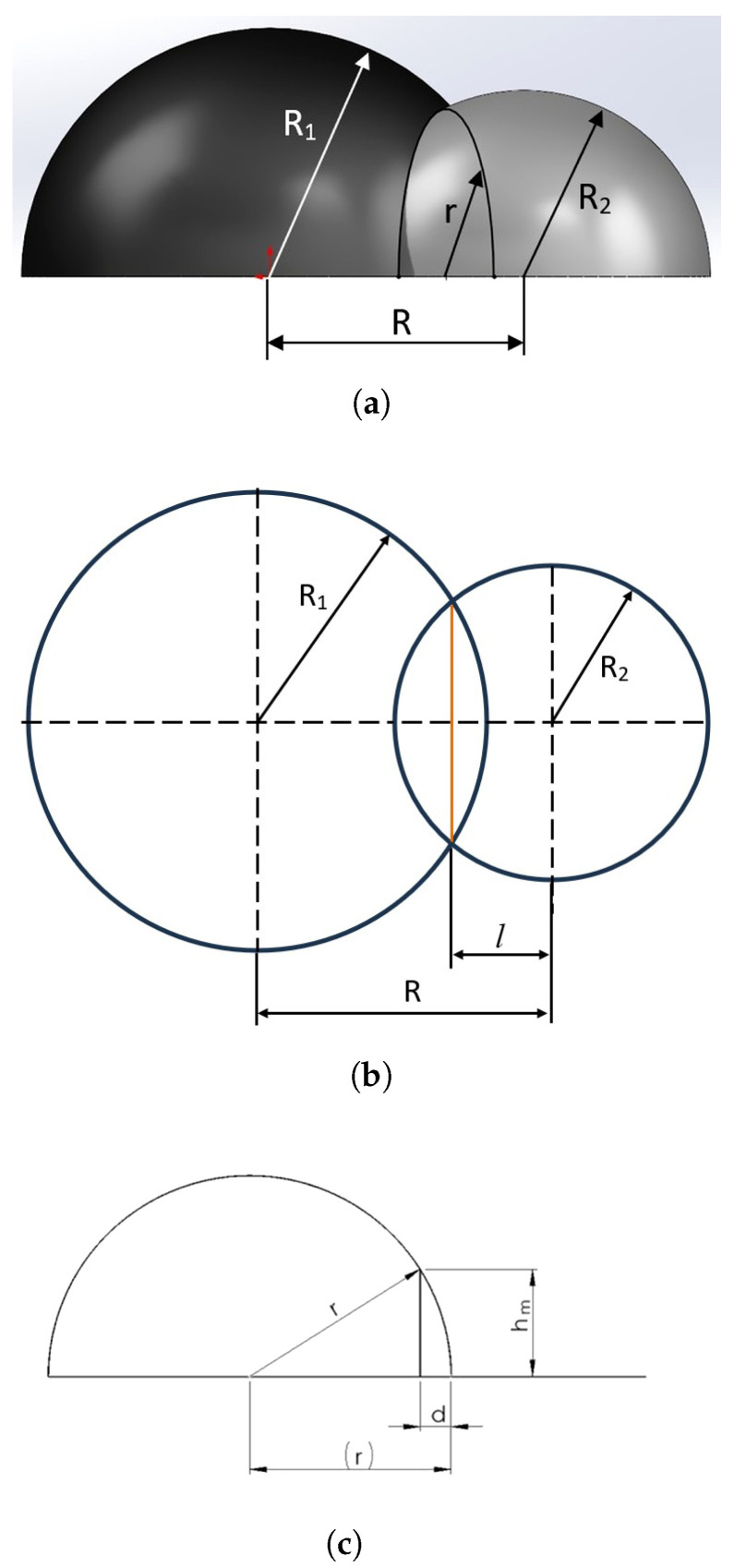
Intersection zone of two hemispheres of possible aircraft location: (**a**) general view of the intersection of the two hemispheres; (**b**) horizontal cross-section showing the intersection of the corresponding circles with radii $R_{1}$ and $R_{2}$; (**c**) vertical cross-section illustrating the semicircular arc representing the possible aircraft location. The orange curve indicates the semicircular arc formed by the intersection of the two spheres, whose radius *r* is shown in subfigure (**c**).

The method of accounting for uncertainty in angular coordinates can be illustrated by two non-directional receiving antennas. After filtering out the fictitious distance data, the remaining real distances are used. Figure 5 shows different cross-sections of the intersection zone of the two hemispheres corresponding to possible aircraft locations. Figure 5a shows the general form of their intersection, Figure 5b shows their intersection in the form of circles in the horizontal plane corresponding to the ground level (assumed to be an ideal flat plane), and Figure 5c shows a side view of the intersection curve describing the possible aircraft location, which has the form of a vertically oriented semicircle.

The geometric relations are given by

(1) $$R_{1}^{2}={\left(R-l\right)}^{2}+r^{2}$$

(2) $$R_{2}^{2}=l^{2}+r^{2},$$

from which the value of *l* is obtained as

(3) $$l=\frac{R^{2}+R_{2}^{2}-R_{1}^{2}}{2R}$$

If $h_{m}$ denotes the maximum height, then according to Figure 5c,

(4) $$r^{2}=h_{m}^{2}+{\left(r-d\right)}^{2},$$

which leads to

(5) $$d^{2}-2rd+h_{m}^{2}=0$$

and hence,

(6) $$d=r\left(1-\sqrt{1-{\left(\frac{h_{m}}{r}\right)}^{2}}\right).$$

Equation (6) is valid for $h_{m}\leq r$.

On this basis, for data processing in an active radio system for aircraft localization and countermeasures, protected against radio direction finding of transmitting modules and consisting of two units using the fictitious electromagnetic center method with two non-directional receiving antennas, the following algorithm can be proposed:

1. All distances to the aircraft determined by the two receiving devices are examined. These distances are the radii of spheres on whose surfaces the detected objects may be located and result from signal reflections in each of the four bistatic channels: ${\mathrm{Tx}}_{1}{\mathrm{Rx}}_{1},\;{\mathrm{Tx}}_{2}{\mathrm{Rx}}_{1},\;{\mathrm{Tx}}_{1}{\mathrm{Rx}}_{2},\;{\mathrm{Tx}}_{2}{\mathrm{Rx}}_{2}$.
2. Pairs of radii whose sum equals a single radius are identified, with the latter being considered fictitious (due to reception through fictitious bistatic channels ${\mathrm{Tx}}_{2}{\mathrm{Rx}}_{1}$ and ${\mathrm{Tx}}_{1}{\mathrm{Rx}}_{2}$. These fictitious radii are eliminated from further analysis.
3. The probable aircraft location is determined in the form of a segment of a vertical semicircle with radius *r*, positioned at a distance from the first or second transceiver antenna, respectively, with this segment having a horizontal projection of length *d*.
4. Based on the determined average possible distances to the aircraft, the phases of the signals arriving at the probable location are modified so that they become successively in phase and out of phase. On the basis of the variation in the signals at the outputs of the correlation receivers, all remaining fictitious distances to the aircraft are eliminated.

If necessary, additional verification can be performed to eliminate false locations, for example, through analysis of the received signal powers in the two channels, evaluation of Doppler components in the receiving centers, analysis of trajectory characteristics of the objects, and other methods, including the use of machine learning algorithms.

## 5. Conclusions

In the present study, a block diagram of a Bistatic Radar with Quantum-Generated Noise Phase Manipulation and Non-Directional Antennas is proposed. The system enables localization of radar-reflective objects with effective suppression of false targets and employs a quantum communication system that provides a practically infinite random digital sequence for controlling phase manipulation. This approach either prevents or introduces significant uncertainty in determining the actual location or direction of the transmitting modules.

The analysis results define the fields of applicability and the limitations associated with the use of this type of bistatic radar, as well as its capabilities for integration into a network of similar bistatic or monostatic radars with overlapping radar surveillance areas.

The proposed approaches can also be applied when using other types of signal manipulation of the transmitted waveforms (e.g., frequency, amplitude, or combined manipulation) and for developing communication systems with continuous full-duplex connectivity.

In practical deployment of the proposed approach, established methods for mitigating random phase errors in bistatic radar systems must be applied. Such errors may be caused by atmospheric turbulence, platform vibrations, multipath propagation, and related effects that degrade coherence.

Principal mitigation techniques include active phase referencing, limiting the coherent processing interval (CPI), and adaptive digital phase compensation [51,52,53,54].

Under relatively stable atmospheric conditions, it can be assumed that for L-band systems, the CPI may reach several hundred milliseconds (beyond which atmospheric and geometric effects become dominant), whereas for X-band systems, the CPI is typically limited to several tens of milliseconds, even with effective tracking.

Consequently, L-band operation allows for a longer CPI and therefore larger bistatic detection zones (up to several hundred kilometers), while X-band operation requires a shorter CPI, resulting in smaller bistatic detection zones (several tens of kilometers) but higher accuracy and spatial resolution.

## 6. Patents

Bistatic Radar with Quantum-Generated Noise Phase Manipulation and Non-Directional Antennas, patent application No. BG/P/2026/114232, filed on 23 January 2026.

## Data Availability

The data that support the findings of this study are available from the corresponding author upon reasonable request.
